# Exploring Mentorship as a Novel Approach to Improving Quality of Life in Sarcoma Survivors: A Qualitative Pilot Study

**DOI:** 10.1155/2021/2042785

**Published:** 2021-08-10

**Authors:** Lotta Våde, Ivar Hompland, Lena Fauske

**Affiliations:** ^1^Sarcoma Patient Organization, Ovre Vollgt. 11, Oslo 0158, Norway; ^2^Department of Oncology, Norwegian Radium Hospital, Oslo University Hospital, P.O. Box 4953 Nydalen, Oslo 0424, Norway; ^3^Department of Interdisciplinary Health Sciences, Institute of Health and Society, University of Oslo, Postboks 1089 Blindern, Oslo 0317, Norway

## Abstract

**Backgrounds:**

To investigate whether a formal mentoring program involving mentors from the business community could improve the quality of life (QoL) of sarcoma survivors struggling with the late effects of treatment.

**Methods:**

Seven former sarcoma patients participated in an eight-month formal mentoring program. The program was assessed through a qualitative study involving a phenomenological approach that utilized a hermeneutical design. In-depth, semistructured interviews were conducted with the mentees after the intervention and six months later. The mentors were interviewed after the program was over. The gathered data were interpreted using a thematic analysis.

**Results:**

The program facilitated dialogue between the mentors and mentees as well as between the mentees. Afterwards, the mentees were more willing to accept the challenges they faced following cancer treatment. During the program, the mentees were pushed out of their comfort zone, which led to mastery and personal growth in them all. However, the program also revealed some additional challenges, including unfulfilled expectations in two mentor-mentee relationships.

**Conclusions:**

The mentoring program facilitated the mentees' reorientation and enhanced their QoL. Its eight-month duration appeared important in terms of allowing the mentees to go through a long-lasting process with continued support. The program could serve as the basis for larger studies involving other cancer survivors.

## 1. Introduction

Sarcoma survivors, who are most commonly adolescents and young adults, frequently struggle with the late effects of treatment [1–3]. Sarcoma often requires intensive multimodal treatment involving extensive surgery as well as both radiation and chemotherapy [4, 5], which all significantly add to the risk of developing long-term therapy-related consequences. Receiving a cancer diagnosis and then undergoing the required treatment are likely to have a major impact on an individual's life. It has been established that a serious disease represents a biographically disruptive event associated with ongoing physical and psychosocial impacts [6] and further that patients often need to reorient themselves and attempt to develop a new identity that is reflective of their “new normal” [7]. This is certainly the case for many sarcoma survivors, who tend to struggle to reorient themselves to the new normal and so require additional guidance [8, 9]. Many of the challenges they face, however, are outside the scope of the normal oncological follow-up process. Thus, there exists a clear need for innovative approaches to studying reorientation and the best means of improving the quality of life (QoL) of cancer survivors [10].

Mentoring is a well-established method for promoting personal development, and it could be a valuable approach for cancer survivors who are struggling with the late effects of treatment. The goal of mentorship is to make the mentee aware of their resources as well as to provide guidance on how to use those. Mentoring often takes the form of a relationship between a more experienced person (the mentor) and a less experienced person (the mentee). The connection between the two parties should be reciprocal, albeit asymmetrical, and it can be useful for both of them, although the primary goal of mentoring is to foster the growth and development of the mentee [11]. The idea of formal mentoring refers to organizationally initiated efforts to match mentors and mentees [12]. This type of mentoring often has defined goals and a specific timeline [13], in addition to following guidelines concerning both interaction frequency and content. Orientation and training are commonly offered to help the mentor and mentee to understand their role obligations and to become comfortable with the mentoring process [12]. Additionally, the mentor should be outside the mentee's chain of command [14]. Studies have found that mentoring can affect the mentee's behavior, attitudes, health, relationships, motivation, and career in a positive way [14, 15].

A number of mentoring programs in which health professionals or peers act as mentors have been studied. For instance, support programs that include former cancer patients mentoring newly diagnosed cancer patients appear to have a positive impact on the satisfaction of the mentee [16, 17]. There are also data confirming that mentoring from health-care professionals can benefit patients [18]. To the best of our knowledge, however, no studies of cancer mentoring programs that involve mentors who are not health-care professionals or peers have previously been reported in the literature. Therefore, Sarkomer, the Norwegian patient association for current and former sarcoma patients, teamed up with health-care professionals working at the sarcoma clinic of the Oslo University Hospital Norwegian Radium Hospital (OUH NRH) to initiate a mentoring program in which sarcoma survivors were provided with mentors from the business community. The rationale behind the development of the program was that working with such mentors would help to focus the mentees' attention on living a healthy and normal life involving friends, family, studies, work, activities, sport, and hobbies, rather than focusing on their illness.

The aim of this qualitative study was to explore whether a formal mentoring program involving mentors from the business community could facilitate the reorientation of sarcoma survivors who were struggling with the late effects of treatment and so help to improve their QoL.

## 2. Methods

This qualitative study adopted a psychosocial perspective on health and illness with the aim of identifying the reasons behind the experienced phenomena, as expressed by the participants themselves. In line with the study's methodological framework as well as the research questions to be addressed, we applied an interpretative phenomenological approach to disease and illness [7, 19]. Phenomenological research aims to investigate individual human experiences (i.e., phenomena) as they manifest in both daily life and specific situations. The field of hermeneutics relates to the methods used to understand and interpret the phenomena of interest in a comprehensible manner [20]. In this case, comprehension is based on both the participant's and the researcher's preunderstandings, as well as on the context in question, and it develops throughout the entire research process [21].

### 2.1. Participants

This retrospective study included 14 participants who took part in the mentoring program: seven sarcoma survivors/mentees and seven mentors. The sarcoma survivors were recruited into the program through information provided via Sarkomer's website (sarkomer.no), social media channels such as Facebook and Instagram, and brochures handed out at the OUH NRH's sarcoma outpatient clinic. All former sarcoma patients were invited to send in an application describing their challenges and motivations, in addition to any personal goals they wanted to achieve through participating in the program. Twelve applications were received. Among them, five potential participants were excluded: two who were considered to be facing significant mental challenges and so were referred back to the health-care system instead, one who experienced a recurrence of sarcoma during the selection process, and two who did not express any goals and so would probably not have benefited from the program. The selection of mentees was carried out by a social worker and a cancer nurse, both with long experience in patient guidance. Six of the seven patients had getting back to work or increase their working time as the main goal. Other goals were to gain a better social life and/or start physical activities. The clinical and epidemiological data of the seven former sarcoma patients are summarized in Table 1. Their self-reported late effects and improvements are presented in Table 2. The mentees are given fictitious names in this study.

To recruit the mentors, we contacted various private companies. The companies were each asked to contribute €4000, half of which would help to finance the program, while the other half would be donated to a charity (FORUT, a Norwegian aid organization). In addition, the companies were each asked to provide a suitable mentor for the program. These mentors came from different backgrounds, but they shared a genuine desire to help another human being. They also believed that this program could increase their knowledge about cancer survivorship and add a new perspective of life. Four mentors were women and three men, aged between 35 and 50. Unlike a regular mentoring program, where the mentor has knowledge and skills in a given field, the mentors in this study were not expected to have any previous experience of cancer survivorship. Two mentors had nurse education, and the other had different positions in human resources, engineering, and economics.

### 2.2. Intervention

The content of different parts of this program was based on experiences from a similar program for cancer survivors in Sweden and the knowledge and experience of a social worker and a nurse. The mentoring program ran for an eight-month period from September 2017 to May 2018. It included three joint eight-hour sessions in which both the mentees and the mentors participated together with two nurses, a social worker, and an oncologist. The sessions involved lectures about sarcoma and long-term side effects with a special emphasis on fatigue, as well as crisis management. These topics were especially important for the mentors because they got an understanding on the challenges the mentees may encounter after sarcoma treatment. In addition, everyone participated in group discussions, and conversations took place between the mentees and mentors. Throughout the entire period of the program, there was individual contact between the mentees and mentors. They were told to be in contact at least once a month. The number of contacts and how they met was an agreement between each dyad, individually tailored to the need of each mentee. During their first meetings, they were asked to establish goals for the cooperation and clarify expectations to each other. The program ended with a one-week trip to Nepal during which the mentees and mentors visited, among other things, a cancer hospital for children and a home for girls who had been sexually abused. This trip was an important part of the program for the mentees to challenge their comfort zone and let them know they could manage to achieve such a long travel. The trip to Nepal was mandatory for the mentees and voluntary for the mentors, although six out of seven of the latter participated. After the program ended, the sarcoma survivors/mentees were invited to deliver a lecture at their specific mentor's workplace concerning their cancer treatment journey as well as their experience of the program. The reason for this lecture was to challenge the mentees and to give the companies an increased insight what it is like to be a sarcoma survivor. The same nurses, social worker, and oncologist that participated on the joint sessions were available to all the mentees and mentors during the meetings and throughout the entire program if they needed guidance.

### 2.3. Procedures

The participants were informed that participation in the study would not affect their oncological follow-up treatment and that they had the option to withdraw from the study at any point. The first author interviewed the mentors at their offices after the intervention was completed, while the last author interviewed the mentees twice: once after the intervention finished and then again six months later to see if any positive changes would last over time. The first interviews with the mentees were all conducted on a face-to-face basis, while the second interviews were either face-to-face or conducted by phone. The average length of the interviews was approximately 45 minutes. The interviews were conducted in accordance with a semistructured framework. They were audio recorded and then transcribed verbatim by a medical secretary.

It is important to disclose that author one worked as a cancer nurse and author two worked as an oncologist, both at the sarcoma department at OUH NRH. They knew some of the participants before the program, but not the mentors. They took part in the whole mentor program, as well as the Nepal trip. Therefore, the last author, who is not a health-care provider and did not know the participants and the program, conducted both the interviews with the mentees and was responsible for the data analysis.

The participants were invited to narrate their story from the point of diagnosis to the present day. In particular, they were requested to describe their life situations as sarcoma survivors prior to the intervention. They were also asked questions concerning their experiences of the mentorship program: How has your everyday life been affected by participating in the mentorship program? How would you describe your relationship with your mentor? How did you experience the trip to Nepal and how did it affect you? What do you think your future holds? (Supplementary Table S1-2).

In the case of the mentors, they were asked the following questions: How have you experienced being a mentor during this project? What is your relationship with your mentee? How do you view your mentee's experiences? What do you think about the program? (Supplementary Table S3). All the gathered information was stored confidentially, and all the transcripts were deidentified.

The data protection officer at the OUH approved the study (approval number 18/07098), and written informed consent was obtained from all the participants.

### 2.4. Data Analyses

The gathered data were analyzed using a reflexive thematic analysis based on the approach of Braun and Clarke [22] to identify patterns of meaning across the dataset in order to answer the research questions. The patterns were identified through a rigorous process of data familiarization, data coding, theme development, and revision. The entire dataset was coded in detail by hand, thoroughly, inclusively, and extensively, by the first and last authors. The codes were then divided into categories, themes, and concepts. The emergent themes formed the core of the analysis. Finally, the themes were assessed in light of the study's objectives and compared with the existing literature and theory [22]. Throughout the whole process of analysis, the researchers regularly returned to the original data to check the themes and quotes in an effort to ensure that the meaning had not been lost during either interpretation or translation [21].

## 3. Results

The mentoring program facilitated dialogue and the exchange of experiences between the mentors and mentees as well as between the seven mentees. We determined that this made the mentees more willing to accept the challenges they face following cancer treatment and in the life they are now living. During the program, the mentees experienced being pushed out of their comfort zone, which led to mastery and personal growth for all of them.

The program also involved a number of challenges, including unfulfilled expectations, challenges in the mentor-mentee relationship, and the long distances between the parties. Both the mentors and mentees agreed that the duration of the program was important and suggested that it should be increased from eight months to one year (Table 3).

### 3.1. Acceptance of the New Conditions

All the mentees reported that their extensive cancer treatment had resulted in late effects that had major impacts on their lives. As Betty put it, “I don't think I can be the way I used to be before I got sick. […] it feels kind of like I have almost completely lost my identity.” The mentees stated that participating in the mentoring program had helped them to accept that neither they nor their life would be the same as before they had cancer. They noted the particular importance of the many talks they had with the mentors and of being in a group with peers who share the same experience and understanding. The mentees' increased self-acceptance meant that they had left various challenges behind and been able to get on with their lives under the new conditions. In this context, it is important to note that the mentors helped the mentees to reverse negative and destructive thought patterns. For instance, Susan's mentor described her experience of Susan's development as follows: “We shifted our focus from concentrating on the challenges and all the negative things to seeing the opportunities.”

The mentoring program demonstrated that the mentees were subject to different conditions than before their treatment, although the program also made them realize that they were just as valuable as they were before. Accepting themselves and their new situation meant that the mentees stopped making an effort to be exactly the same as prior to having cancer. This helped them to relax and let go of feelings such as shame and worthlessness. As Chris said, “But still, in fact, I can do most of what other people can do, maybe it takes me more effort, but at the same time I have some things that many other people don't have. I can see things from another angle, which other people can't.”

It was difficult for many of the mentees to accept that they did not have as much energy as they did before. Several of them pushed themselves too hard, despite the fact that they had little energy. Half of the mentees were, therefore, encouraged by their mentors to accept that they could not be as active as they used to be. Furthermore, they were told that it was perfectly alright to take things easy. Helen said, “I can accept more now, including just lying down on the couch. I accept that I'm not worth less than others even though I need to rest.” The mentees also stated that they could now take breaks without experiencing a guilty conscience.

Many mentees reported having often felt alone with their thoughts and feelings for many years after the end of their treatment. Being with others who faced the same everyday experiences and challenges helped to increase their understanding of what people normally experience following cancer treatment, which made them less critical of themselves. Susan stated “I think we all got the feeling that we weren't alone. […] and kind of feeling 100% normal again, that was really amazing.” Moreover, Janet said “It helped a lot to accept the situation.” Such acceptance made it easier for the mentees to be open about their situation at work and their experiences in social settings. They realized that what they had previously been ashamed of was actually a normal consequence of the treatment they had undergone. They also reported that the program had equipped them with words they could use to explain their situation to others. As Susan noted, “It's all about communication, finding good ways to explain things.” All the mentors confirmed these findings. For instance, John's mentor said “Whatever thoughts he's had, he may have felt that they were particular to him, but then, it turned out that many of his thoughts and challenges were the same as those experienced by the others.”

The findings during the first round of interviews concerning the mentees' increased acceptance of who they were and the life they were now living became even more evident during the second rounds of interviews some six months later. The majority of them felt that greater self-acceptance was the most positive change they experienced as a result of the mentoring program, and they found that the change lasted long after the program ended.

### 3.2. Pushed out of the Comfort Zone

All the mentees reported having experienced a sense of mastery and personal growth because they had been pushed out of their comfort zone. They had previously been uncomfortable or afraid when faced with the challenges associated with participating in the program. As they had been closely followed up by their mentors and because they were part of a group of peers, they found that they were actually comfortable when outside of their comfort zone.

The majority of mentees initially found it frightening to open up to a stranger. However, this feeling soon dissipated, and all the mentees felt that it was good to talk to their mentors. Janet's mentor said “We had an open dialogue, very open and honest. It was a trusting relationship from day one.” The mentees found that they were pleased to talk to a person who knew nothing about them and who was not a family member, friend, or health professional. They felt that they could talk more freely. Furthermore, it was easier to open up and talk about their challenges. Betty described her experience in the following way:

“You know, it's been a […] really incredible experience to dare to have a close relationship, to open up to people you don't know, and to get that support […], to feel the effect of it, I'd never have believed it, you really have to experience it for yourself.”

The mentors underlined the importance of the mentees relating their stories to someone from the outside who could see everything with new eyes. Indeed, Betty's mentor likened it to her mentee's story being front-page news again.

The mentees had different challenges that they wanted to work on during the program, for example, getting a job or restarting their social life. After over a decade of cancer, Sara began her first job when she was middle-aged: “My first job [...] going to work, I felt so great. Absolutely beautiful. But still, when I face challenges, I'm simply terrified.” She commented that it was reassuring to have a mentor who supported her and gave her good advice: “Sometimes, I was really dreading something [...] so then I could call my mentor. She managed to push me and told me not to give up.” Sara reflected that her mentor's support was crucial in terms of helping her to keep her job, which was important to her sense of mastery and personal growth. John's goal was to resume his social life, which had proved challenging due to his pain and disability. He also had a poor self-image. John explained that his mentor pushed him to contact his friends and family and then followed up on it in a supportive manner. “Then, he'd check that I'd done it and that was fine because then I just had to go off and visit people.” Many mentees recognized the value of having a mentor who pushed them to make agreements and then checked on them afterwards. This meant that they followed through on performing the agreed activities more frequently and, thus, achieved the goals they had set. This was confirmed by John's mentor: “When he was pushed to make contact, it didn't take long before a friend called him and asked if he wanted to join them. That was something he described as almost unimaginable before (the mentoring program).”

All the mentees challenged themselves to try new activities or to resume activities they had not engaged in for a long time. They reported still being physically active six months after completing the program. The mentees were also good at motivating each other to join in activities. For instance, two mentees started to exercise in a pair. The mentors and mentees collaborated in planning a joint relay race for a year later, despite the mentees' major physical challenges. Here, the group stood together to set a goal that they also achieved.

The most challenging activity for most of the mentees, which pushed them way outside of their comfort zone, was the trip to Nepal. One participant, Betty, had actually decided in advance not to go on the trip: “I'd actually signed up for the mentoring and thought I'd drop the trip because it was scary and, well, it was just too much.” They all had various concerns before the trip. They were afraid of diseases, bacteria, pain, or being unable to complete the whole program due to fatigue. It was also challenging to spend a week with people they did not know very well. In particular, the long flight would be difficult for those with major disabilities. However, their concerns turned out to be unjustified. The trip exceeded all the mentees' expectations and they were highly satisfied. Betty described the trip as follows:

“So, it was an absolutely amazing experience, and well, as I said, all about mastery. Just to travel a long way and to be able to join in with all the activities there, every single day, having enough energy for that.”

The experience of mastering challenging situations boosted the mentees' confidence, and they all felt tougher and more daring after the trip. John said “But now, it's like I have an alarm clock in my head that tells me I can do much more than I think I can. I mean, if I could do that trip, well, then I can do a lot more things at home too.”

The visit to the home for girls who had been victims of sexual abuse as well as the trip to the pediatric cancer ward left a strong impression on the mentees and gave them new perspectives on their own situation. Susan said “That was a trip to put things into perspective, and like so many of the others said afterwards, my God, our lives are absolutely great. We're very lucky, despite what we've been through. […] and […] this extreme realization of what the world is like and how lucky we are to be living in Norway and getting treatment here.”

Most of the mentees stated that the reassurance of having their mentor with them and the support provided by the group gave them a feeling of mastery throughout the trip. During the second round of interviews, all the mentees confirmed that they were no longer afraid to venture outside of their comfort zone. Chris said “I can still feel the sense of mastery. I'm always stepping a bit outside of my comfort zone, so yes, that's a good feeling.” They found that they were tougher and capable of setting boundaries and standing up for themselves. Their self-confidence had improved, and many had set new goals for the future.

Most of the mentors stated that one of the main dilemmas they faced was determining how much they could push their mentee outside of his or her comfort zone. Fatigue was an unfamiliar experience for the mentors, and they saw how it constrained the mentees' everyday lives. However, none of the mentees reported that they had been pushed too hard.

### 3.3. Experiences of the Mentoring Program

All the mentees felt that it was important to have their mentor available to them throughout the program. None of the mentors found this to be a burden. Sara's mentor said “It really benefits me a lot to be used for something I'm good at, something that doesn't drain me, but something I look forward to and get something out of.”

The mentors had all volunteered to take part in the program, and they received no payment for their participation. They signed up because they wanted to give something of themselves and to be part of something they considered meaningful. Janet's mentor explained “I wanted to develop an alternative perspective to my work perspective, which was pretty much focused on business and the hard side of life, so I wanted to see a different dimension and focus a bit more on people and actually be able to help someone.”

The mentors felt that they had learned a lot about themselves during the program and gained new perspectives on life. As Susan's mentor pointed out, “I feel that this program has strengthened my existential preparedness. […] I'm very grateful that I've been part of this.”

Mentoring was new to both the mentors and the mentees. The two groups had different expectations and thoughts regarding what they could contribute, which led to various challenges. For instance, John's initial mentor was replaced because he did not maintain regular contact with his mentee: “First I got a mentor who didn't help me at all. He never called me. I was the one who had to contact him all the time, which I didn't think that was the idea. And he suggested things I wasn't really interested in.” Conversely, one mentor was frustrated because her mentee did not want to meet her as often as she had expected. Betty's mentor said “She doesn't answer the phone. Also, I tried to meet her several times, but it was never suitable.” One mentee, Janet, stated that having a mentor of the opposite sex rather inhibited communication. She said “Maybe it would have been easier for me if [my mentor] was a woman. I thought about that afterwards, not while we were in contact. I think I might have been even more open.” Several mentees felt that living a long way from their mentor proved challenging for their relationship. John and his mentor lived far from each other, and John emphasized “My mentor could have lived closer so we could have met for a cup of coffee […]. It's important to have real contact with people, not just over the phone.” Several participants felt it was very important that arrangements were made for the mentors and mentees to meet on a face-to-face basis.

### 3.4. Suggestions to Improve the Program

This program was a pilot, and some experiences were made that could be considered in future programs. The feedback from both mentors and mentees gave us several inputs on areas that could be improved.

All the participants (both mentees and mentors) underlined the importance of the mentoring program lasting for eight months. To achieve lasting change, it was necessary for the process to be a long one. The mentors had to repeat their messages many times throughout the program. Susan was constantly reminded by her mentor of the importance of feeling good about herself:

“You're active and you can actually give a lot of yourself, in spite of your fatigue. And she drummed that into my head many times, so I felt it as a kind of confidence boost.”

The majority of mentees and mentors wished that the program lasted even longer, for example, for a whole year. They wanted the program to continue and have one more meeting after the trip to Nepal.

There were other challenges associated with the program, including finding private companies that had employees who wanted to volunteer as mentors and who lived in the same areas as the mentees. In our program, we tried to match the mentors and mentees in terms of their locations, interests, and genders. In cases where we did not achieve this, challenges did arise, especially for the mentees. In the future, it is important to work to overcome such challenges and better meet the needs of the mentees.

Several mentors highlight the importance of including mentees who are motivated and prepared to make an effort to reach their goals. Chris's mentor expressed “I think the value increases if they are challenged harder on the accepted entrance.” To achieve a functioning cooperation between the mentor and mentee, the mentors stated that they need to have the same expatiations to the process of their collaboration. To make sure this happens, future programs should have more strict criteria to be included in the program and that the staff should keep closer contact to both mentors and mentees, especially the first months of the program.

The program had confidentiality rules that were found to be too demanding. This made it difficult for the mentors to discuss challenges they experience with their mentee, with other mentors in the program. They believe it limited the collaboration between the mentors and the possibility to learn and support each other. Betty's mentor explains: “We should be allowed to talk to each other because it could have made it a little easier, in relation to frustrations which both mentors and participants can feel.” In the future program, confidentiality should be maintained, but the program could have some new guidelines inside the group. This could give the possibility for both mentors and mentees to talk more openly to other members of the program.

After eight months, the program was completed and the formal obligations to both mentor and mentee stopped. Still, some mentors and mentees explained that they felt insecure about whether the contact could be maintained or not. The frames of future programs should make sure they all know what to expect when the program is over.

## 4. Discussion

To the best of our knowledge, this study is the first to explore a formal mentoring program in cancer survivors where the mentor is neither a peer nor a health-care provider. Our findings indicate that the QoL of sarcoma survivors who are struggling with fatigue, disabilities, pain, a changed identity, and reduced self-image, as a consequence of their cancer treatment, can be enhanced by a mentoring program involving mentors from the business community. In such a program, the focus is on healthy and normal relationships with family and friends and participation in work and other activities, as well as self-image. The mentorship program seemingly resulted in a better QoL for the mentees, including increased self-acceptance regarding who they had become following their cancer treatment and feeling comfortable moving outside of their comfort zone. This led to better coping in everyday life as well as greater faith in themselves and how they could contribute and participate. They could now set new goals they had not dreamed of before joining the mentorship program.

The mentees emphasized that accepting themselves and the fact that their life had changed following their cancer treatment was the most important outcome of the mentoring program. Being cured of cancer does not necessarily mean returning to the life you lived prior to falling ill. For many, recovery from cancer means starting a new life based on different conditions [23]. Patients who have been affected by a serious illness and its consequences often have to reorient their lives and construct a new identity based on changed assumptions [7, 24, 25]. Helping the mentees to understand and accept these changed assumptions was a key focus of the mentors in this program. The fact that the mentors commented on how the mentees had now accepted their situation and ceased to strive to be who they once were showed that the mentees had changed their view of themselves and what they could achieve. In line with other research [8, 26], our findings show that turning adversity into something positive or into personal growth is useful for those undergoing reorientation after cancer treatment. Moreover, the interactions with other sarcoma survivors who had had similar experiences also helped the mentees to normalize their situation. Normalization has been identified as a common coping strategy among individuals living with multiple conditions [27], while normalization in the face of disruption may be an important process for cancer patients [6]. These findings show that the increased self-acceptance led to less feelings of shame and worthlessness for the participants.

A mentor's task might involve making their mentee aware of their strengths and resources and helping them to see opportunities, which will facilitate the growth and development of the mentee [11]. All the mentees in this study confirmed that they had experienced increased coping and personal growth through participating in the mentorship program. They had been pushed outside of their comfort zone in several regards, although they experienced it as safe due to the support of their mentor and the group.

It is easy to stagnate in the safe and the familiar when life becomes challenging and daily chores seem insurmountable, when your body works against you, the pain disturbs your sleep, and the fatigue never goes away. The mentors' task included challenging this state of affairs. One advantage the mentors had was that they did not know the mentees prior to the program. It proved demanding for the mentees to open up to a stranger, but it also proved useful to talk to someone from the outside, who had not closely observed all their challenges and who looked at them with fresh eyes. The challenges they were supported in addressing included restarting a social life with family and friends as well as focusing on working life and increased participation in various activities. The mentors focused on prompting the mentees to look forward, seize opportunities, and break barriers. The mentors noted that the mentees all too frequently placed limitations on themselves due to fatigue, pain, and impairment, although through participating in the mentorship program, they came to feel that the unknown and impossible were possible. In fact, the mentees were stimulated to do the unthinkable. In particular, the trip to Nepal showed them how much they could still do. During the trip, they ventured far outside of what they had defined as their comfort zone, and they found that doing so went very well. Both the mentors and the mentees expressed gratitude for the opportunity to participate in the program, and they emphasized that they had gained new and valuable perspectives on life through doing so.

All the mentees commented that they struggled with chronic fatigue, the most common and arguably most troublesome long-term side effect following cancer treatment [28–30]. Fatigue is largely unrecognized in clinical practice [31, 32], and research indicates that a change of approach is required so that fatigue is treated as central to patient management both during and after systemic cancer treatment [31]. Our findings show that self-acceptance and a focus on opportunities rather than on problems, in addition to having help from a supportive person who motivated them to stay focused over time, provided the mentees with energy and a sense of peace with their life. Moreover, when they experienced how going outside of their comfort zone did not drain them of energy or cause additional problems in their everyday life, such an approach actually increased their QoL.

In line with prior research [12], some challenges were also identified in relation to the mentoring program, including mentor neglect (by one mentee), unmet expectations (by one mentor and one mentee), geographical separation from the mentor (by several mentees), and feelings of personal inadequacy (by several mentors). The mentors also expressed that it was difficult to know how hard they could push the mentees when they themselves did not have knowledge or experience of fatigue. They were afraid that they might reverse the positive progress due to having no knowledge of the mentees' limits. However, the fact that none of the mentees felt pushed too far may indicate that a mentor with no knowledge of cancer survivorship can overcome such concerns within a structured framework.

The current study did have some limitations. First, the small sample size may limit the generalizability of the findings. Second, the selection process used in this study introduced a bias toward a group of sarcoma survivors who were particularly struggling following treatment. Yet, in qualitative research, one does not seek to gather representative data, but rather to elucidate the phenomena that the participants experience based on their own perspectives. We conducted a retrospective pilot study on this program to explore the impact of the intervention and the participants' experiences. Therefore, no interviews were conducted at baseline, which would have elicited expectations for the program and observed how these changed over time. Another important limitation to acknowledge is that this was a convenience sample, the authors cannot be sure that saturation was reached, but the selection proved to be adequate in this study, as the gathered narratives were rich and full of nuanced examples. Lastly, we must acknowledge that a mentorship program, as seen in the present study, is time and resource consuming, which means that it might be difficult to implement on a larger scale. However, for those who are young and particularly those who are struggling to return to their work, social life, and other activities, there are unfortunately no quick fixes. A program such as the one implemented in this study could facilitate reorientation and so has a major positive impact on the QoL of cancer survivors.

## 5. Conclusions

In this study, the formal cancer survivorship mentoring program involving mentors from the business community assisted with reorientation and improved the QoL of the participating sarcoma survivors. We believe that it is key to the success of this kind of intervention that the mentees enter into a long-lasting process with continued support to overcome their challenges. The mentorship program explored in this study could serve as the basis for larger studies involving other groups of cancer survivors.

## Figures and Tables

**Table 1:** Mentees' demographic information and disease characteristics (*N* = 7).

Demographic and disease characteristics	No.
Sex (male:female)	2 : 5
*Age* ^*1*^	
20–35	4
36–50	2
51–60	1

*Marital status*	
Married	1
Cohabitant	1
Single	5

*Parental status*	
Children	4
No children	3

*Employment status*	
Student	1
Full employment	1
Partly employed/disability benefit	4
Benefit recipient	1

*Time since finished treatment*	
2 years	4
4–5 years	2
>10 years	1

*Type of sarcoma*	
Ewing sarcoma	2
GIST	1
Chordoma	1
Rhabdomyosarcoma	1
MPNST	1
Chondrosarcoma	1

*Treatment*	
Surgery	3
Surgery + chemotherapy	1
Surgery + chemotherapy (including HMAS)	2
Surgery + chemotherapy + radiotherapy	1

^1^Age when enrolled in the mentorship program. HMAS = high-dose chemotherapy with stem-cell rescue; GIST = gastrointestinal stromal tumor; MPNST = malignant peripheral nerve sheath tumor.

**Table 2:** Self-reported challenging late effects and improvements described by the mentees (*N* = 7) in the applications and/or during the interview.

Late effects before participating in the mentorship program	No.
Functional impairment	4
Fatigue	7
Pain	4
Feeling of shame	3
Feeling inferior	5
Reduced self-esteem	6
Reduced emotional state/mood	6

*Improvements after participating in the mentorship program*	
Increased acceptance of new situation	7
Less afraid	4
Increased time at work	4
Started charity work	3
Feeling less shame	5
Increased self-esteem	7
Increased emotional state/mood	7

**Table 3:** Codes and themes extracted from the participants' interview.

Themes	Acceptance of the new conditions	Pushed out of the comfort zone	Experiences of the mentoring program
Selected codes	(i) Mentor helped me to accept the new situation (ii) Envisioning more opportunities rather than limitations (iii) Discovered metaphors/ways to explain things (iv) There is nothing wrong with me, rather a natural consequence of the treatment (v) The things I struggle with is a lot more common than I thought and recognizing this helped, indeed, to accept my situation (vi) I am not worthless even if I need to rest (vii) Less self-critical (viii) I am as valuable as other people (ix) Life has more value than I thought (x) Less shame (xi) I am more accepting of my tiredness and being exhausted (xii) I am not alone with my thoughts and challenges	(i) More self-confident and secure (ii) Experience being able to do more than I thought I could (iii) Mentor pushed me not to give up working (iv) Amazing experience to have the courage to be more open, let people closer to you, and receive support (v) More open and honest in relationships (vi) More open to explore new avenues without worrying about things that could go wrong (vii) Really stepped out of my comfort zone (viii) Dare to do more, less scared of conflicts, and give more attention to myself (ix) Want to help, I am now a volunteer with the Red Cross (x) I have challenged myself to travel. It went very well. I am proud of myself (xi) Reconnected with friends (xii) Learned to hold on to my social life (xiii) Challenged to be a bit tougher, take more space, and fight more (xiv) I was not sure how hard I could push the mentee	(i) Important not to live too far from the mentor (ii) Wish the program could last for one year (iii) It is important to clarify expectations between the mentor and mentee (iv) Would have profited from a closer connection between me and the mentor (v) It would have been better to ensure more openness if the mentor was a woman like me (vi) It is preferred to meet the mentor in person (vii) I am grateful for the opportunity to be a mentor in this program (viii) I have learned a lot about myself being a mentor related to personal development, perspectives on life, and faith in humanity

## Data Availability

The datasets used in this study are not publicly available due to patient confidentiality issues, although they are available from the corresponding author upon reasonable request.

## Supplementary Materials

Supplementary Materials Supplementary Table S1: interview guide mentees conducted after having finished the mentorship program. Supplementary Table S2: interview guide mentees conducted six months after completing the program. Supplementary Table S3: interview guide for the mentor conducted after having finished the mentorship program.

## References

[1] Winnette R., Hess L. M., Nicol S. J., Tai D. F., Copley-Merriman C. (2017). The patient experience with soft tissue sarcoma: a systematic review of the literature. *The Patient - Patient-Centered Outcomes Research*.

[2] Longhi A., Ferrari S., Tamburini A. (2012). Late effects of chemotherapy and radiotherapy in osteosarcoma and Ewing sarcoma patients: the Italian Sarcoma Group experience. *Cancer*.

[3] Barrera M., Teall T., Barr R., Silva M., Greenberg M. (2012). Health related quality of life in adolescent and young adult survivors of lower extremity bone tumors. *Pediatric Blood and Cancer*.

[4] Casali P. G., Bielack S., Abecassis N. (2018). Bone sarcomas: ESMO-PaedCan-EURACAN Clinical Practice Guidelines for diagnosis, treatment and follow-up. *Annals of Oncology: Official Journal of the European Society for Medical Oncology*.

[5] Casali P. G., Abecassis N., Aro H. T. (2018). Soft tissue and visceral sarcomas: ESMO-EURACAN Clinical Practice Guidelines for diagnosis, treatment and follow-up. *Annals of Oncology: Official Journal of the European Society for Medical Oncology*.

[6] Bury M. (1982). Chronic illness as biographical disruption. *Sociology of Health & Illness*.

[7] Svenaeus F. (2011). Illness as unhomelike being-in-the-world: heidegger and the phenomenology of medicine. *Medicine, Healthcare & Philosophy*.

[8] Fauske L., Bondevik H., Ahlberg K., Bjørndal A. (2019). Identifying bone sarcoma survivors facing psychosocial challenges. A study of trajectories following treatment. *European Journal of Cancer Care*.

[9] Zambrano S. C., Kollár A., Bernhard J. (2020). Experiences of return to work after treatment for extremital soft tissue or bone sarcoma: between distraction and leaving the disease behind. *Psycho-Oncology*.

[10] Quinn G., Goncalves V., Sehovic I., Bowman M., Reed D. (2015). Quality of life in adolescent and young adult cancer patients: a systematic review of the literature. *Patient Related Outcome Measures*.

[11] Eby L. T., Rhodes J. E., Allen T. D., Allen T. D., Eby L. T. (2007). The blackwell handbook of mentoring. *The Blackwell Handbook of Mentoring: A Multiple Perspectives Approach*.

[12] Eby L. T., Lockwood A. (2005). Protégés’ and mentors’ reactions to participating in formal mentoring programs: a qualitative investigation. *Journal of Vocational Behavior*.

[13] Murray M. (2002). *Beyond the Myths and Magic of Mentoring: How to Facilitate an Effective Mentoring Process*.

[14] Eby L. T. (1997). Alternative forms of mentoring in changing organizational environments: a conceptual extension of the mentoring literature. *Journal of Vocational Behavior*.

[15] Eby L. T., Allen T. D. (2008). Moving toward interdisciplinary dialogue in mentoring scholarship: an introduction to the special issue. *Journal of Vocational Behavior*.

[16] Geiger A. M., Mullen E. S., Sloman P. A., Edgerton B. W., Petitti D. B. (2000). Evaluation of a breast cancer patient information and support program. *Effective Clinical Practice: ECP*.

[17] Amin A. L., Neuner J., Duthie E. A., Finn V. R., Kong A. L. (2014). A one-to-one mentoring support service for breast cancer survivors. *Wisconsin Medical Journal: Official Publication of the State Medical Society of Wisconsin*.

[18] Pini S. (2009). Education mentoring for teenagers and young adults with cancer. *British Journal of Nursing*.

[19] Kvale S., Brinkmann S. (2009). *Interviews: Learning the Craft of Qualitative Research Interviewing*.

[20] Edmonds W. A., Kennedy T. D. (2017). *An Applied Guide to Research Designs: Quantitative, Qualitative, and Mixed Methods*.

[21] Gubrium J. F., Holstein J. A., Marvasti A. B., McKinney K. D. (2012). *The SAGE Handbook of Interview Research: The Complexity of the Craft*.

[22] Braun V., Clarke V. (2006). Using thematic analysis in psychology. *Qualitative Research in Psychology*.

[23] Drew S. (2007). `Having cancer changed my life, and changed my life forever’: survival, illness legacy and service provision following cancer in childhood. *Chronic Illness*.

[24] Frank A. W. (1995). *The Wounded Storyteller: Body, Illness, and Ethics*.

[25] Parsons J. A., Eakin J. M., Bell R. S., Franche R.-L., Davis A. M. (2008). So, are you back to work yet? Re-conceptualizing “work” and “return to work” in the context of primary bone cancer. *Social Science & Medicine*.

[26] Park C. L., Chmielewski J., Blank T. O. (2010). Post-traumatic growth: finding positive meaning in cancer survivorship moderates the impact of intrusive thoughts on adjustment in younger adults. *Psycho-Oncology*.

[27] Sanderson T., Calnan M., Morris M., Richards P., Hewlett S. (2011). Shifting normalities: interactions of changing conceptions of a normal life and the normalisation of symptoms in rheumatoid arthritis. *Sociology of Health & Illness*.

[28] Bower J. E. (2014). Cancer-related fatigue-mechanisms, risk factors, and treatments. *Nature Reviews Clinical Oncology*.

[29] Berger A. M., Mooney K., Alvarez-Perez A. (2015). Cancer-related fatigue, version 2.2015. *Journal of the National Comprehensive Cancer Network*.

[30] Mitchell S. A. (2010). Cancer-related fatigue: state of the science. *PM&R*.

[31] Koornstra R. H. T., Peters M., Donofrio S., Van den Borne B., De Jong F. A. (2014). Management of fatigue in patients with cancer-a practical overview. *Cancer Treatment Reviews*.

[32] Hompland I., Fauske L., Lorem G. F., Bruland Ø. S. (2020). Use of a simple form to facilitate communication on long-term consequences of treatment in sarcoma survivors. *Clinical Sarcoma Research*.

