# Supplementary material for: Exploring Mentorship as a Novel Approach to Improving Quality of Life in Sarcoma Survivors: A Qualitative Pilot Study
Source: Sarcoma. 2021 Aug 10;2021:2042785. doi: 10.1155/2021/2042785 (PMC8371637; doi:10.1155/2021/2042785)
Supplement: Supplementary Materials — Supplementary Table S1: interview guide mentees conducted after having finished the mentorship program. Supplementary Table S2: interview guide mentees conducted six months after completing the program. Supplementary Table S3: interview guide for the mentor conducted after having finished the mentorship program. [file 2042785.f1.docx]

| Tell your story | From when you first became ill, received your diagnosis and how life has been and still is until today |
| --- | --- |
| Perception of your life situation. What can you tell us about living with long term side effects after sarcoma treatment? |  |
| Why did you apply for participation in the mentorship program? |  |
| How is your everyday life now affected by your participation in the mentorship program? |  |
| How would you describe your relationship with your mentor during the program? |  |
| How did you experience being with others having received treatment for sarcoma? | How has it affected you? |
| How has your participation in the mentorship program affected: - Tell | - Your physical activity  - Your energy in daily life  - Your work situation / everyday study / everyday life  - Your immediate family  - Belief in yourself (self-confidence)  - Your social life  - Your free time  - Other |
| How did you experience the trip to Nepal? | How has the journey affected you? |
| What do you think about the future? | - Do you still have contact with your mentor? What does it mean? If no, why not?  - Do you think your participation in the mentorship program will affect your further life? - Tell |
| What kind of expectations did you have for the mentorship program? | How were they fulfilled? |
| Is there anything you would like to mention that I haven’t asked about? |  |

**Table I** Interview guide mentees conducted after having finished the mentorship program

**Table II** Interview guide mentees conducted six months after completing the program

| How would you describe your life now as compared to before the program and right after the program ended? | Social life  Job / volunteer work  Activities  Travel / mastery / out of the comfort zone  Acceptance of own situation |
| --- | --- |
| What is the best / most important thing you have gained from the mentorship program? |  |
| How do you now see your future compared to before the mentorship program? |  |
| How has contact with the mentor been after the program has ended and what do you think about the future? |  |
| How has the contact with the others in the group been after the program has ended and how do you think it will be in the future? |  |
| Is there anything you would like to mention that I haven’t asked about? |  |

Codes identified from the first interview as a basis for the second interview

| D1 | D2 | D3 | D4 | D5 | D6 | D7 |
| --- | --- | --- | --- | --- | --- | --- |
| Fatigue  Anxiety disease  Emotional stress  The self-image | Fatigue  Social Media  New things | Acceptance  Calm down  Nepal Travel  Stronger | Pain  View of life (worthwhile) | Acceptance  Mindset  Possibilities/  restrictions  Get out | Fatigue  Acceptance  Normalcy | Fatigue  Normalcy  Acceptance  Adjust your own expectations  Pondering |

**Table III** Interview guide for mentor conducted after having finished the mentorship program

| Why did you agree to be a mentor in the mentorship program? |  |
| --- | --- |
| Have you been a mentor before? In case, in what context? |  |
| What kind of long-term side effects did you learn that your participant had? |  |
| How would you describe your relationship with your participant? |  |
| How do you think the mentorship program has affected the everyday life of your participant? |  |
| How do you experience your participant’s experience being with others who have been treated for sarcoma? |  |
| How do you feel that the mentorship program has affected the following of your participant: Tell | - Physical activity  - The energy of daily life  - His / her work situation / everyday study / everyday life  - His / her immediate family  - Belief in yourself (self-confidence)  - His / her social life  - His / her free time  - Other |
| How did you experience your mentoring role during the trip to Nepal? |  |
| How did you experience that the trip to Nepal affected your participant? |  |
| Do you still have contact with your participant? | What does it mean? If no, why not? |
| Do you think participating in the mentorship program will affect the future for your participant? |  |
| How have you experienced being a mentor in this program? |  |
| What kind of expectations did you have for the mentorship program? | How were they fulfilled? |
| Is there anything you want to tell / share that I have not asked about, that you think is important? |  |
